# Robotic Surgery from a Gynaecological Oncology Perspective: A Global Gynaecological Oncology Surgical Outcomes Collaborative Led Study (GO SOAR3)

**DOI:** 10.3390/diseases13010009

**Published:** 2025-01-06

**Authors:** Faiza Gaba, Karen Ash, Oleg Blyuss, Dhivya Chandrasekaran, Marielle Nobbenhuis, Thomas Ind, Elly Brockbank, on behalf of the GO SOAR Collaborators

**Affiliations:** 1 Department of Gynaecological Oncology, University College Hospital, University College London Hospitals NHS Foundation Trust, London NW1 2BU, UK; 2 Institute of Applied Health Sciences, University of Aberdeen, Aberdeen AB24 3FX, UK; 3 Aberdeen Royal Infirmary, NHS Grampian, Aberdeen AB25 2ZN, UK; 4 Wolfson Institute of Population Health, Queen Mary University of London, London EC1M 6BQ, UK; 5 Department of Paediatrics and Paediatric Infectious Diseases, Institute of Child’s Health, Sechenov University, 119435 Moscow, Russia; 6 Department of Gynaecological Oncology, The Royal Marsden Hospital, London SW3 6JJ, UK; 7 Department of Gynaecological Oncology, The Royal London Hospital, Barts Health NHS Trust, London E1 1FR, UK

**Keywords:** robotic surgery, gynaecological oncology, learning curve, situational awareness, green theatres

## Abstract

Background/Objectives: For healthcare institutions developing a robotic programme, delivering value for patients, clinicians, and payers is key. However, the impact on the surgeon, training pathways, and logistics are often overlooked. We conducted a study on the impact of robotic surgery on surgeons, access to robotic surgical training, and factors associated with developing a successful robotic programme. Method: In our international mixed-methods study, a customised web-based survey was circulated to gynaecological oncologists. The Wilcoxon rank-sum test and Fisher’s exact test, tested the hypothesis of the differences in continuous and categorical variables. Multiple linear regression was used to model the effect of variables on outcomes adjusting for gender, age, and postgraduate experience. Outcomes included situational awareness, surgeon fatigue/stress, and the surgical learning curve. Qualitative data were collected via in-depth semi-structured interviews using an inductive theoretical framework to explore access to surgical training and logistical considerations in the development of a successful robotic programme. Results: In total, 94%, 45%, and 48% of survey respondents (n = 152) stated that robotic surgery was less physically tiring/mentally tiring/stressful in comparison to laparoscopic surgery. Our data suggest gender differences in the robotics learning curve with men six times more likely to state robotic surgery had negatively impacted their situational awareness in the operating theatre (OR = 6.35, *p* ≤ 0.001) and 2.5 times more likely to state it had negatively impacted their surgical ability due to lack of haptic feedback in comparison to women (OR = 2.62, *p* = 0.046). Women were more risk-averse in case selection, but there were no self-reported differences in the intra-operative complication rates between male and female surgeons (OR = 1, *p* = 0.1). In total, 22/25 robotically trained surgeons interviewed did not follow a structured curriculum of learning. Low and middle income country centres had less access to robotic surgery. The success of robotic programmes was measured by the number of cases performed per annum, with 74% of survey respondents stating that introducing robotics increased the proportion of surgeries performed by minimal access surgery. There was a distinct lack of knowledge on the environmental impact of robotic surgery. Conclusions: Whilst robotic surgery is considered a landmark innovation in surgery, it must be responsibly implemented through effective training and waste minimisation, which must be a key metric in measuring the success of robotic programmes.

## Simple Summary

There has been a rapid integration of robotics into gynaecology surgical practice; however, the impact on the surgeon, training pathways, and logistics of set-up are often overlooked. We conducted a study on the impact of robotic surgery on gynaecological oncology surgeons, access to robotic surgical training, and factors associated with developing a successful robotics programme. Our data indicate robotic surgery is less physically demanding in comparison to laparoscopic surgery. The data also show gender differences in the acquisition of non-technical skills in the learning curve of robotic surgery, international disparities in access to robotics, and a lack of awareness of the environmental impact. Whilst robotic surgery is a landmark innovation in surgery, it must be responsibly implemented through effective training and waste minimisation, which must be a key metric in measuring the success of a robotic programme.

## 1. Introduction

The use of robotic surgery is continuing to increase in gynaecological oncology, as robotic platforms allow surgeons to perform more precise procedures for patients with complex conditions [1,2,3]. The ergonomic advantages of robotic surgery are most apparent during the performance of complex surgical tasks with lower physical stress reported [3,4]. Whilst there has been much focus on impact of robotics on the musculoskeletal system of the operating surgeon, there is a paucity of data in relation to the impact on cognitive ergonomics and situational awareness. In addition, there are no data on gender differences.

As more gynaecological cancer centres are adopting robotic surgery, fellowship training programmes have started incorporating robotics into their curriculum of teaching [5]. Whilst this has broadened surgical training, there are limited data on the quantification of the effects of robotic surgery on learning curves in gynaecological oncology or access to robotic training [5].

The increasing need for and dissemination of this technology associated with the high costs requires careful planning during its implementation. The success and duration of a robotic surgery programme is dependent upon its long-term results [6]. Success may be defined from many different perspectives. For example, the increase in proportion of surgical cases performed via minimal access surgery since the introduction of robotics, the number of trained robotic surgeons, or even theatre carbon footprint, as there is a shift towards green theatres with net zero carbon [7].

We present data from an international mixed methods study on (1) the impact of robotic surgery on gynaecological oncology surgeons; (2) access to robotic surgical training; (3) factors associated with developing a successful robotic programme.

## 2. Materials and Methods

We sent an anonymised web-based survey in English to gynaecological oncology members of the European Network of Young Gynae Oncologists (ENYGO), the Society of Gynecologic Oncology (SGO), the Society of European Robotic Gynaecological Surgery (SERGS), and the British Gynaecological Cancer Society (BGCS) between February and November 2023. Individuals (trainees and consultants/attendings) performing gynaecological oncology surgery robotically were eligible.

For data analysis, duplicates were excluded with the first entry, and all incomplete questionnaires were included. The thirty-five item questionnaire (File S1) evaluating the impact of robotic surgery on gynaecological oncologists incorporated situational awareness; physical/mental fatigue; stress; musculoskeletal injuries; tactile/haptic feedback; learning curve; operating time; and complications.

### 2.1. Questionnaire Development

An initial hard copy draft was developed following the literature review. Each question was systematically discussed and reviewed by ten gynaecological oncology clinicians (from the UK/US/India/Italy). On the basis of expert opinions, each item was given a relevance score from 1 (least relevant) to 4 (most relevant). The scores were reviewed, low relevance items deleted, and questionnaire length was optimised to facilitate compliance. A pilot amongst five clinicians was undertaken for usability, technical functionality, and layout, with feedback incorporated.

### 2.2. Statistical Analysis

Descriptive statistics were calculated for the baseline characteristics. The Wilcoxon rank-sum test and Fisher’s exact test were used for testing the differences between the continuous and categorical variables. Given the distribution of our data, the Wilcoxon rank-sum test was utilised for comparing medians between groups, as it does not assume normality. For categorical variables, Fisher’s exact test was selected, due to its reliability in analysing smaller sample sizes such as the subgroups in our study.

Multiple linear regression was used to model the effect of variables on situational awareness, surgeon fatigue/stress, tactile feedback, learning curve, and operating time. Multiple analyses were adjusted for gender, age, and postgraduate experience. Two-sided *p* values are reported for all statistical tests. Statistical analysis was performed using R version 3.5.1.

### 2.3. Qualitative Data

Qualitative data (collected between February 2023 and May 2024) were collected via in-depth semi-structured one-to-one interviews with robotic surgical teams conducted face-to-face, virtually, using a pre-developed topic guide. The development of the topic guide was informed by literature review and expert consultation (File S2), evaluating access to robotic surgical training and the factors associated with developing a successful robotic programme. All interviews were audio recorded and transcribed verbatim. Robotic surgeons, anaesthetists, and nurses were invited. Surgeons from institutions without a robotics programme were also invited. The topics covered included access to robotic surgical training, theatre efficiency, and surgical waste production. The transcripts were analysed using an inductive theoretical framework, and the data were managed using NVIVO-v12. Two researchers (FG/KS) independently coded the transcripts, following a three-step process (open, axial, and selective coding). Researchers (FG/KS) visited centres performing live robotic surgery to collect observational qualitative data. Analysis was performed in parallel with the data collection and finalised once theoretical saturation was achieved.

## 3. Results

In total, 152 participants from 35 countries responded to the electronic survey. Using the World Bank Index classification, 23 (15%) were from 12 low and middle income countries (LMIC), with 129 (85%) from 24 high income countries (HIC, Table S1). The baseline characteristics are displayed in Table 1. In total, 64% (97/152) of the respondents were consultants/attendings, and 36% (55/152) were trainees (fellows/residents). In addition, 56% (85/152) were male, and 44% (67/152) female. 74% (111/150) stated the introduction of robotic surgery had increased the uptake of minimal access surgery (MAS) in their centre overall.

In total, 32% (49/152) strongly agree/agree that robotic surgery has had a negative impact on intra-operative situational awareness (defined as the perception of elements in the environment, comprehension of their meaning, and the projection of their status in the near future) when compared to laparoscopic surgery (Table S2). Men were six times more likely than women to state that robotic surgery had negatively impacted their situational awareness (OR = 6.35, *p* ≤ 0.001). However, individuals with a greater number of years of robotics experience and those performing increasing numbers of robotic surgeries per annum were less likely to state a negative impact (OR = 0.893, *p* = 0.029; OR = 0.98, *p* = 0.04, Table 2). The most common reasons given for reduced situational awareness included the surgeon sitting at the console away from the surgical team (80%, 39/49), reduced visual perception (73%, 36/49), and impaired communication with the operating room (55%, 27/49, Table S3).

In total, 26% (39/150), strongly agree/agree surgical ability is negatively impacted by the lack of haptic feedback (Table S4). Men were 2.5 times more likely to state a negative impact on surgical ability in comparison to women (OR = 2.62, *p* = 0.046). But individuals with increasing years of robotics experience and those performing greater numbers of robotic surgeries per annum were less likely to state a negative impact (OR = 0.91, *p* = 0.04; OR = 0.96, *p* = 0.04, Table 3).

In total, 1% (2/144) stated the intra-operative complications during robotic surgery in comparison to laparoscopic surgery were higher/somewhat higher, with 68% (98/144) the same and 31% (44/144) lower/somewhat lower (Table S5). The reasons cited for more complications included the absence of haptic feedback (2/2) and less experience using the robotic surgical platform (2/2). There was no difference in self-reported complication rates between male and female surgeons (*p* = 0.1)

In total, 94% (137/146) stated robotic surgery was very much/somewhat less physically tiring in comparison to laparoscopic surgery (Table S6). In addition, 45% (66/146) and 48% (69/145) stated robotic surgery was very much/somewhat less mentally tiring and stressful in comparison to laparoscopic surgery (Tables S7 and S8). Tables S9 and S10 indicate that levels of mental fatigue and stress both reduced with increasing number of robotic surgeries performed per annum (OR = 0.965, *p* = 0.041; OR = 0.967, *p* = 0.038). In total, 32% (48/148) of respondents had taken time off work due to a musculoskeletal injury sustained during laparoscopic surgery with the mean number of days off being 3.4 (SD = 3.2, range = 0–14) and 1.9 (SD = 0.9, range = 1–4) for consultants and trainees, respectively. In comparison, 0.7% (1/147) of respondents had sustained a musculoskeletal injury necessitating sick leave whilst performing robotic surgery.

For the total operating time (including docking and the surgical procedure but excluding anaesthetic time), 50% (72/144) strongly agree/agree that robotic surgery takes longer than laparoscopic surgery (Table S11). Here, 50% (36/72) stated surgeon speed, 93% (67/72) stated time associated with docking, and 31% (22/72) stated an inexperienced wider surgical team as reasons for increased operating time (Table S12). Consultants had shorter operating times than trainees (OR = 0.213, *p* = 0.014,) as well as surgeons performing a larger number of cases per annum (OR = 0.969, *p* = 0.003, Table S13).

For the rate of conversion to laparotomy, 2.1% (3/144) stated robotic surgery was higher/somewhat higher, 72% (103/144) the same and 26.4% (38/144) somewhat lower/lower in comparison to laparoscopic surgery (Table S14).

In total, 7.2% (11/152) stated the learning curve with robotic versus laparoscopic surgery was much steeper/steeper, with 7.9% (12/152) the same and 85% (129/152) less steep/much less steep (Table S15). Individuals with a greater number of years of laparoscopic surgery experience (OR = 0.791, *p* = 0.014) and those performing a larger number of laparoscopic cases per annum (OR = 0.798, *p* = 0.044) had a less steep learning curve (Table 4).

In total, 89% (136/152) stated that they prefer robotic surgery over laparoscopic surgery. The most commonly cited reasons (Table S16) included greater surgical precision (114/136), enhanced visualisation of the surgical field (113/136), and less physical exhaustion (111/136). 11% (16/152) stated they do not prefer robotic over laparoscopic surgery. The most commonly cited reasons (Table S17), included less experience with robotic surgery (8/16) and the lack of haptic feedback (6/16).

Thirty individuals (twenty-five surgeons, three nurses, and two anaesthetists) were interviewed from seven countries. Of these, 8/25 surgeons interviewed were fellows/residents, 13/30 women, and 8/30 from LMIC settings. The interviews lasted 60–75 (mean = 66) minutes. Surgeons from institutions without a robotic programme did not accept our invitation to be interviewed. Three categories were elicited from the collected data: individual, organizational, and national.

### 3.1. Individual

Within this category, three themes emerged: learning curve, ergonomics, and gender. All surgeons interviewed stated their learning curve was faster with robotic in comparison to laparoscopic surgery because they were already experienced in open/laparoscopic surgery and so not having to learn the surgical procedure. Instead, they were learning to use new surgical technology/equipment. Additionally, dual-console training sped up the learning curves. However, not all centres had access. Trainees (fellows/residents) stated their learning curves were slowed in centres where their consultants/attendings were robotically naive. In such circumstances, the trainees were bedside assistants for longer before being allowed onto a console. Improved surgical ergonomics was a recurrent theme with all surgeons reporting less musculoskeletal strain with robotic surgery in comparison to laparoscopic/open surgery. Gender differences were apparent. Women were more risk-averse and cautious in case selection in terms of case complexity, particularly in earlier stages of their learning curve, whereas men were more likely to attempt more complex surgeries earlier on. In addition, men struggled with situational awareness early on in their learning curve and felt disadvantaged by not being at the bedside. However, both situations resolved as the number of cases performed by the surgeons increased.

### 3.2. Organisational

Two themes emerged: theatre efficiency and environment. Robotic theatre efficiency was maximised by ensuring surgeons were console trained before setting-up a robotics programme, thereby ensuring the robot was in constant use. Training the nursing team simultaneously with surgeons ensured smooth running of lists with the entire team able to troubleshoot. To facilitate communication and prevent a negative impact on situational awareness, the placement of the console in theatre in a position where the surgeon was able to visualise and hear both the anaesthetist and bedside assistants was important. Theatre turnover (time between cases) was optimised by having the same team performing robotic surgery. The console surgeon constantly communicating with the bedside assistants and scrub team (who intra-operatively have less to do in comparison to open/laparoscopic surgery) during the procedure kept the team focused on the operation, and they were less likely to be distracted. This was particularly important during critical steps or an intra-operative complication, ensuring all team members remained focused. Interviewees reported that the success of robot programmes was measured by hospital management by annual procedure volumes, robot utilization, and the number of trained robotic surgeons, whilst from the clinician’s perspective, success was measured by post-operative morbidity and the number of days spent as an inpatient in hospital. Only 3/30 interviewees were aware of the environmental impact of robotic surgery and consciously made decisions to minimise waste.

### 3.3. National

Two themes emerged: accreditation and disparities in access to MAS. 22/25 interviewed surgeons (who were all experienced open/laparoscopic surgeons) underwent informal training with a proctor/mentee being present for early cases with some also undergoing wet lab or simulation training. Only 3/25 had followed a structured programme and were accredited by international robotic societies. LMIC centres faced additional challenges in establishing robotics. Firstly, many surgeons had not developed MAS skills and were accustomed to primarily open surgery. Secondly, insurance companies would not cover the cost of an operation being performed robotically. Thirdly, the cost of buying a robot and the associated maintenance fees were too high.

## 4. Discussion

In total, 94%, 45%, and 48% of survey respondents stated robotic surgery was less physically tiring, mentally tiring, and stressful, respectively, in comparison to laparoscopic surgery. Our results suggest gender differences in robotic learning curves, with men six times more likely to state robotic surgery had negatively impacted their situational awareness (OR = 6.35, *p* ≤ 0.001) and 2.5 times more likely to state that the lack of haptic feedback had a negative impact on their surgical ability in comparison to women (OR = 2.62, *p* = 0.046). However, the negative impact of both lessened with increasing robotic surgery experience. Women were more risk averse in case selection. Access to robotic surgical training was facilitated by the presence of dual consoles during training cases, as well as trainers that were already proficient in robotic surgery. In total, 22/25 robotically trained surgeons interviewed did not follow a structured curriculum of learning and were not accredited. LMICs had less access to robotic surgery. Factors associated with the development of a successful robotics programme include surgeons who were console trained ahead of establishing a programme; simultaneous training of the entire theatre team; careful positioning of console in the operating room optimising situational awareness and communication; the same team performing robotic surgery; and constant dialogue between the console surgeon and the wider surgical team to maintain engagement. The success of robotic programmes was measured by the number of cases performed per annum, with 74% of survey respondents stating that establishing a robotic programme increased the proportion of surgeries performed by MAS. There was a distinct lack of knowledge on the environmental impact of robotic surgery and waste minimisation.

In keeping with the published literature [8,9,10], our quantitative data associated less physical discomfort and fewer musculoskeletal injuries in surgeons with robotic surgery in comparison to laparoscopic surgery. The reported rate of musculoskeletal injuries amongst general surgeons ranges between 50 and 85% [11,12]. A large survey of American vascular surgeons found that up to 6% of respondents had retired early as a result of musculoskeletal injuries [11]. As per the published data, our results demonstrate robotic surgery is associated with less cognitive fatigue and stress for the surgeon [13,14]. Our data indicate that robotic surgery may preserve career longevity by protecting physical health and reducing cognitive fatigue/stress.

Gender differences have been widely investigated in high stress environments (aeronautics, nuclear facilities), where it is vital to system safety. Gaze movement is a surrogate marker of situation awareness, with studies showing women have more gaze movements between necessary information elements than men [15,16]. This is consistent with our findings of improved situational awareness amongst female robotic surgeons. Whilst the lack of effective haptic feedback is often reported by surgeons and robotics researchers to be a major limitation of current robotic platforms [17], ours is the first data showing that this affects male surgeons more than female surgeons. However, our data suggest that both situational awareness and adjusting to the loss of haptic feedback are skills that can be learned and improve with increasing experience. Women self-reported being more risk averse, particularly in the earlier stages of their learning curve, but there were no gender differences in intra-operative complications. However, as complication rates were self-reported, they may not have been accurate. A population based retrospective cohort study of one million Canadian adults undergoing surgery showed that patients treated by female surgeons had lower rates of adverse post-operative outcomes including death at 90 days and 1 year after surgery compared with those treated by male surgeons, citing careful case selection as one potential reason [18]. Robotic training curriculums must include the assessment of non-technical skills (situational awareness, case selection, and insight), and trainers must be aware of gender differences when training trainees to support effective learning.

Our qualitative data indicate that many established open/laparoscopic surgeons have undertaken robotic surgery without following a standardised validated robotic curriculum. However, it is not possible to compare the learning curves of experienced surgeons and surgically naive trainees who will be learning the steps of the operation and how to safely use a new surgical device simultaneously. Hence, there is a need for competency based training programmes led by surgical colleges [19]. For example, a measurable indicator of an adequately trained robotic surgeon would be one who had successfully completed recognised training implemented through a structured curriculum, such as that offered by the British and Irish Association of Robotic Gynaecological Surgeons (BIARGS) or the Society of European Robotic Gynaecological Surgery (SERGS). Future research comparing surgeon proficiency following curriculum based organised accreditation versus informal instruction would objectively highlight differences in approaches. Key metrics for measurement could include post-operative morbidity, situational awareness, and surgeon confidence. The published data indicate that patients treated by surgeons judged to have a high level of technical skill have better outcomes than those with a lower skillset [20]. This is of particular relevance, as robotic platforms are increasingly used to perform more complex operations, hence the need for operative performance indicators (OPIs) as benchmarks for competent surgeons [21]. Our data indicate that dual console access for improving learning curves is important, both for trainers and institutions, and may improve access to robotic surgery.

Our quantitative and qualitative data indicate international disparities in access to robotic surgery with LMIC settings being adversely affected. In the GO SOAR1 study mapping international variations in post-operative morbidity and mortality, MAS was found to be an independent variable in reducing intra-operative and minor post-operative morbidity [22]. However, LMICs versus HICs were statistically significantly more likely to undergo open surgery (68.9%, 40.5%, *p* ≤ 0.001) than MAS (20.9%, 52.8%, *p* ≤ 0.001) [22]. Published data suggest that MAS may be safe, effective, feasible, and cost-effective in LMICs, although it often remains limited in its accessibility, acceptability, and quality [23,24,25]. Surgeons, policy makers, and manufacturers should focus on plans for the sustainability, training, and retention of MAS providers in LMICs [22,23].

Our data on factors associated with the development of a successful robotics programme are in keeping with published literature [6,26,27]. Robotic surgery programmes are an excellent microcosm of surgical practice within which development of various protocols for training, credentialing, and monitoring are vital [26]. Best practices can be instituted without burdening the system by appropriate division of responsibilities and accountability within centres [26]. Increasing the proportion of surgeries performed via a minimal access route, whilst providing safe and effective patient care, with minimal physical discomfort to the surgeon and minimising surgical waste production must be the ultimate goal. MAS has a high environmental cost. With global temperatures rising and unmet surgical needs persisting, there is much interest in carbon and material footprints of surgeries and strategies to make MAS greener. Only 3/30 interviewees were familiar with the term “green theatres”, which is an initiative aimed at reducing the carbon footprint and enabling more environmentally sustainable surgical care. Data on the lack of consideration of environmental factors in surgery has been previously published [28,29]. This is a particularly pressing issue in robotics, as procedures result in 44% higher greenhouse gas emissions and 24% higher waste production than laparoscopy [28]. The increased environmental impact of robotic surgery may not sufficiently offset the clinical benefit and poses an ethical dilemma [28]. Utilizing reusable equipment, repackaging, surgeon preference cards, and increasing staff awareness on open and unused equipment and desflurane avoidance are measurable indicators for reducing greenhouse gas emissions and waste, making robotic surgery more environmentally friendly, which must be a key consideration to establish a successful robotic programme [28].

The study strengths include that this is the first mixed methods international study reporting the impact of robotic surgery specifically on gynaecological oncology surgeons, access to robotics in LMICs, and learning curves. The limitations include the small sample size of 152 survey respondents, thus limiting the clinical inferences and selection bias. The survey was available in only English, therefore excluding non-English speakers, and was only available online to individuals who were members of BGCS/SGO/ENYGO/SERGS. In addition, much of the survey was self-reported, with subjective responses to situational awareness, haptic feedback, and learning curves. Surgeons not performing robotic surgery were not interviewed, and so the interviewed cohort may not sufficiently identify all barriers to establishing a robotic surgical programme.

It is the responsibility of surgical colleges to ensure validated structured training curriculums for safely training robotic surgeons of the future. Trainers must be cognisant of potential gender differences in non-technical skill acquisition and offer targeted training to optimise the learning curves. Environmental waste production must be a key consideration when setting up robotic programmes, ensuring individual surgeon and patient outcome gains are not offset by detrimental environmental consequences that devastate millions of people worldwide, often disproportionately in LMICs, which have less access to robotic surgery. Access to robotic surgery for LMICs must increase to avoid increasing surgical disparities globally. A future economic evaluation study evaluating the cost-effectiveness of robotic surgery in LMICs would contribute data in understanding under what conditions it may be cost-effective in austere environments.

## 5. Conclusions

Data show gender differences in the acquisition of non-technical skills in the learning curve of robotic surgery, international disparities in access to robotic surgery, and a lack of awareness of the environmental impact. Whilst robotic surgery is a landmark innovation in surgery, it must be responsibly implemented through effective training and waste minimisation, which must be a key metric to measure the success of a robotic programme.

## Figures and Tables

**Table 1:** Baseline demographics of survey respondents.

	Job Title		*p* Value
	Consultant (n = 97, %)	Trainee (n = 55, %)	
**WBI**			
LMIC	20 (20.6%)	3 (5.5%)	0.017
HIC	77 (79.4%)	52 (94.5%)	
**Healthcare sector of work**			
Government/state funded	74 (76.3%)	49 (89.1%)	0.122
Private	15 (15.5%)	5 (9.1%)	
Government/state funded and private	8 (8.2%)	1 (1.8%)	
**Mean age (SD, range)**	45.4 (8, 31–65)	37.2 (4.5, 30–60)	<0.001
**Gender**			
Male respondents	69 (71.1%)	16 (29.1%)	<0.001
Female respondents	28 (28.9%)	39 (70.9%)	
**Mean years of postgraduate experience (SD, range)**	17.4 (8.4, 2–35)	9.9 (3.4, 2–16)	<0.001
**Mean years of laparoscopic surgical experience (SD, range)**	13.5 (6.5, 1–29)	6.4 (2.5, 2–16)	<0.001
**Mean operative laparoscopic cases per year as primary/lead surgeon (SD, range)**	45.7 (41.1, 2–160)	31.8 (44.7, 5–300)	0.01
**Mean years of robotic surgical experience (SD, range)**	4.7 (4.6, 1–30)	2.6 (2.2, 1–13)	0.007
**Mean robotic cases for per year as primary/lead surgeon (SD, range)**	33.2 (35.4, 1–200)	25.6 (44.3, 2–275)	0.022
**Mean surgical gynaecological oncology cases per year of cohort (SD, range)**	484.1 (317, 30–1600)		
**Mean (%) surgical case load of cohort as per surgical modality (SD, range)**			
Robotic	27.5 (18.5, 2–80)		
Laparoscopic	25.6 (20.4, 0–85)		
Open	46.9 (18.8, 5–90)		
**Mean (%) surgical case load of centre as per gynaecological cancer primary (SD, range)**			
Endometrial	62.5 (27.1, 5–100)		
Cervical	7.4 (13.9, 0–75)		
Ovarian	4.4 (7.5, 0–40)		
**In your centre, has the introduction of robotic surgery increased the uptake of minimal-access surgery overall?**			
Yes	111 (74%)		
No	39 (26%)		

WBI—World Bank Index; LMIC—low- and middle-income country; HIC—high-income country.

**Table 2:** Multiple regression analysis evaluating impact of variables on situational awareness.

Variable	OR	95% CI	*p* Value
Age	0.962	0.857 to 1.069	0.485
Gender	6.353	2.505 to 17.555	<0.001
Job	0.947	0.278 to 3.117	0.93
Post graduate experience	1.089	0.971 to 1.228	0.15
Laparoscopic surgical experience	1.039	0.931 to 1.161	0.491
Laparoscopic cases per annum	0.992	0.977 to 1.006	0.28
Robotic surgical experience	0.893	0.762 to 1.017	0.029
Robotic cases per annum	0.975	0.95 to 0.996	0.041

N = 124. Strongly agree/agree versus strongly disagree/disagree. Gender: male versus female. Job: consultant versus trainee. Age, post graduate experience, laparoscopic surgical experience (years): linear, laparoscopic cases per annum: linear, robotic surgical experience (years): linear, robotic cases per annum: linear.

**Table 3:** Multiple regression analysis evaluating impact of variables on haptic feedback.

Variable	OR	95% CI	*p* Value
Age	0.899	0.79 to 1.008	0.085
Gender	2.622	1.035 to 6.948	0.046
Job	1.943	0.606 to 6.433	0.267
Post grad experience	1.139	1.007 to 1.298	0.420
Laparoscopic surgical experience	1.021	0.914 to 1.144	0.712
Laparoscopic cases per annum	0.994	0.981 to 1.007	0.408
Robotic surgical experience	0.908	0.774 to 1.034	0.039
Robotic cases per annum	0.961	0.933 to 0.986	0.040

N = 125. Strongly agree/agree versus strongly disagree/disagree. Gender: male versus female. Job: consultant versus trainee. Age, post graduate experience, laparoscopic surgical experience (years): linear, laparoscopic cases per annum: linear, robotic surgical experience (years): linear, robotic cases per annum: linear.

**Table 4:** Multiple regression analysis evaluating impact of variables on learning curve.

Variable	OR	95% CI	*p* Value
Age	0.933	0.768 to 1.099	0.447
Gender	0.69	0.176 to 2.754	0.589
Job	3.543	0.583 to 29.13	0.185
Post grad experience	1.097	0.925 to 1.31	0.287
Laparoscopic surgical experience	0.791	0.848 to 1.173	0.014
Laparoscopic cases per annum	0.798	0.978 to 1.015	0.044

N = 152. Steeper/somewhat steeper versus somewhat less/less. Gender: male versus female. Job: consultant versus trainee. Age, post graduate experience, laparoscopic surgical experience (years): linear, laparoscopic cases per annum: linear.

## Data Availability

Relevant anonymized data can be obtained upon reasonable request from the corresponding author.

## Supplementary Materials

The following supporting information can be downloaded at https://www.mdpi.com/article/10.3390/diseases13010009/s1. Table S1: Survey respondents per country. Table S2: Impact of robotic surgery on situational awareness in comparison to laparoscopic surgery. Table S3: Reasons for reduced situational awareness. Table S4: Impact of lack of haptic feedback on robotic surgical ability in comparison to laparoscopic surgery. Table S5: Intra-operative complications robotic versus laparoscopic surgery. Table S6: Impact of robotic versus laparoscopic surgery on physical fatigue. Table S7: Impact of robotic versus laparoscopic surgery on mental fatigue. Table S8: Impact of robotic versus laparoscopic surgery on stress. Table S9: Multiple regression analysis evaluating impact of variables on mental fatigue. Table S10: Multiple regression analysis evaluating impact of variables on stress. Table S11: Impact of robotic versus laparoscopic surgery on operating time. Table S12: Reasons for increased operating time. Table S13: Multiple regression analysis evaluating impact of variables on operating time. Table S14: Impact of robotic versus laparoscopic surgery on rate of conversion to open surgery. Table S15: Impact of robotic versus laparoscopic surgery on learning curve. Table S16: Reasons survey respondents prefer robotic surgery over laparoscopic surgery. Table S17: Reasons survey respondents do not prefer robotic surgery over laparoscopic surgery. File S1: Survey on impact of robotic surgery on gynaecological oncologists. File S2: Topic guide.
