# Supplementary material for: Robotic Surgery from a Gynaecological Oncology Perspective: A Global Gynaecological Oncology Surgical Outcomes Collaborative Led Study (GO SOAR3)
Source: Diseases. 2025 Jan 6;13(1):9. doi: 10.3390/diseases13010009 (PMC11765035; doi:10.3390/diseases13010009)
Supplement: Supplementary file 1 [file diseases-13-00009-s001.zip › File S1.pdf]

Survey on Impact of Robotic Surgery on Gynaecological Oncologists

Dear Colleague

We would be grateful if you could answer the following survey to try and ascertain the impact of robotic surgery on Gynaecological Oncologists.

This survey is for individuals who are either Gynaecological Oncologists or undergoing Gynaecological Oncology training who perform robotic surgery. This survey is part of a wider process evaluation study on the integration of robotic surgery into routine practice and impact on surgical teams, communication, collaboration, decision making as well as the facilitators and barriers to setting up a robotic service. Please be assured that all responses will be anonymous and individuals will not be identifiable through their participation/responses. Participating in our survey will take no longer than five minutes of your time.

Many thanks for your collaboration.

Dr Faiza Gaba on behalf of GO SOAR (Global Gynaecological Oncology Surgical Outcomes Collaborative)

Please complete the following questions about yourself and clinical practice setting

Q2 **Job Title**

Consultant.................. ☐ Trainee/Fellow .... ☐

Q3 **Gender**

Male .... ☐ Female .... ☐

Transgender male .... ☐ Transgender female .... ☐

Gender variant-non conforming........... ☐ Other .... ☐

Other, please specify

[ ]

Q4 **Age (years)** [ ]

Q5 **How many years of postgraduate experience do you have?** [ ]

Q6 **How many years of laparoscopic surgical experience do you have?** ☐

Q7 **How many operative laparoscopic cases (excluding diagnostic laparoscopies) for oncology do you perform per year as primary/ lead surgeon?** ☐

Q8 **How many years of robotic surgical experience do you have?** ☐

Q9 **How many robotic cases for oncology do you perform per year as primary/lead surgeon?** ☐

Q10 **In total, how many surgical gynaecological oncology cases per year does your centre undertake (includes total number of open, laparoscopic, robotic, vaginal cases)?** ☐

Q11 **Which type of healthcare sector you work in? *(tick all that apply)***

Government/State funded .... ☐ Private .... ☐

Q12 **Hospital type**

University/Academic........................... ☐ District General .... ☐

Please state, what proportion (%) of your surgical cases are :

Q13 **Robotic** ☐

Q14 **Laparoscopic** ☐

Q15 **Open** ☐

Q16 **In your centre, has the introduction of robotic surgery increased the uptake of minimal access surgery overall?**

Yes .... ☐ No .... ☐

Please state, what proportion (%) of your robotic surgical cases take place for the following gynaecological cancers :

Q17 **Endometrial** ☐

Q18 **Cervical** ☐

Q19 **Ovarian** ☐

The following questions are to understand the impact of robotic surgery on your surgical practice in the operating theatre

Q20 **In comparison to laparoscopic surgery, my intra-operative situational awareness (defined as the perception of elements in the environment, the comprehension of their meaning, and the projection of their status in the near future) with robotic surgery is reduced**

- Strongly agree ☐
- Agree ☐
- Neither agree or disagree ☐
- Disagree ☐
- Strongly disagree ☐

Q21 **Reasons for reduced situational awareness *(tick all that apply)***

- Surgeon sits at a console away from the patient and the rest of the operating room team ☐
- Reduced visual perception (i.e. less able to pick up on non-verbal visual cues in the operating room) ☐
- Impaired communication with the operating room team ☐
- Other ☐

Other, please specify

☐

Q22 **When compared to laparoscopic surgery, my surgical ability is negatively impacted with robotic surgery by lack of tactile information/haptic feedback**

- Strongly agree ☐
- Agree ☐
- Neither agree or disagree ☐
- Disagree ☐
- Strongly disagree ☐

Q23 **In comparison to laparoscopic surgery, my intra-operative complications with robotic surgery are**

- Higher ☐
- Somewhat higher ☐
- The same ☐
- Somewhat lower ☐
- Lower ☐

Q24 **Reasons for higher intra-operative complication rate *(tick all that apply)***

- Lack of tactile information/haptic feedback ☐
- Less experience with robotic when compared to laparoscopic surgery ☐
- Other ☐

Other, please specify

[ ]

Q25 **In terms of physical fatigue, robotic surgery when compared to laparoscopic surgery is**

- Very much less physically tiring ☐
- Somewhat less physically tiring ☐
- As physically tiring ☐
- Somewhat more physically tiring ☐
- Very much more physically tiring ☐

Q26 **In terms of mental fatigue, robotic surgery when compared to laparoscopic surgery is**

- Very much less mentally tiring ☐
- Somewhat less mentally tiring ☐
- As mentally tiring ☐
- Somewhat more mentally tiring ☐
- Very much more mentally tiring ☐

Q27 **In terms of stress, robotic surgery when compared to laparoscopic surgery is**

- Very much less stressful ☐
- Somewhat less stressful ☐
- As stressful ☐
- Somewhat more stressful ☐
- Very much more stressful ☐

Q28 **Have you ever had to take time of work (sick leave) due to a musculoskeletal injury sustained whilst performing laparoscopic surgery?**

- Yes ☐
- No ☐

Q29 How long (in days) did you take off? [ ]

Q30 **Have you ever had to take time of work (sick leave) due to a musculoskeletal injury sustained whilst performing robotic surgery?**

- Yes ☐
- No ☐

Q31 How long (in days) did you take off? [ ]

Q32 **In terms of total operating time (time period includes docking as well as surgical procedure but excludes anaesthetic time), my robotic surgical cases take longer than laparoscopic surgery**

- Strongly agree ☐
- Agree ☐
- Neither agree or disagree ☐
- Disagree ☐
- Strongly disagree ☐

Q33 **Reasons for increased surgical time (tick all that apply)**

- Surgeon speed slower with robotic surgery ☐
- Increased time associated with set up and docking of ports ☐
- Operating team less experienced with robotic surgery ☐
- Other ☐

Other

[ ]

Q34 **My rate of conversion to open surgery with robotic surgery when compared to laparoscopic surgery is**

- Higher ☐
- Somewhat higher ☐
- The same ☐
- Somewhat lower ☐
- Lower ☐

Q35 **Reasons for increased conversion rate (tick all that apply)**

- Less experience with managing intra-operative complications robotically ☐
- It takes longer to manage a complication robotically ☐
- Patient cases undergoing robotic surgery are surgically more complex ☐
- Other ☐

Other

[ ]

Q36 **My learning curve for robotic surgery when compared to laparoscopic surgery was**

- Much steeper ☐
- Steeper ☐
- The same ☐
- Less steep ☐
- Much less steep ☐

Q37 **If cost or theatre access were not an issue, for patients suitable for minimal access surgery, would you prefer robotic over laparoscopic surgery**

- Yes ☐
- No ☐

Q38 **Reasons you would prefer robotic surgery (tick all that apply)**

- Less physical exhaustion for the surgeon ☐
- Less mental exhaustion for the surgeon ☐
- Enhanced visualisation in 3D of the surgical field ☐
- Greater surgical dexterity ☐
- Greater surgical precision ☐
- Less reliance on a surgical assistant ☐
- Fewer distractions when operating ☐
- Other ☐

Other

____

Q39 **Reasons you would NOT prefer robotic surgery (tick all that apply)**

- Less experience with robotic surgery ☐
- Negative impact on communication in the operating theatre ☐
- Negative impact on team work in the operating theatre ☐
- Lack of tactile information/haptic feedback ☐
- Spatial configuration of equipment in the operating theatre ☐
- Other ☐

Other

____

Q40 **Any other comments?**

____

Thank you for taking the time to complete this questionnaire
Click the Submit button below to finish
