# Supplementary material for: Robotic Surgery from a Gynaecological Oncology Perspective: A Global Gynaecological Oncology Surgical Outcomes Collaborative Led Study (GO SOAR3)
Source: Diseases. 2025 Jan 6;13(1):9. doi: 10.3390/diseases13010009 (PMC11765035; doi:10.3390/diseases13010009)
Supplement: Supplementary file 1 [file diseases-13-00009-s001.zip › File S2.pdf]

Topic guide for GO SOAR3

**Research Aims to explore**<br>Access to robotic surgery training.<br>Impact of robotic surgery on theatre efficiency<br>Impact of robotic surgery on surgical waste production

1. **Introduction**
- Introduce self, funder.
- Purpose of the research.
- Introduce audio recorder.
- Stress all identifiable information will be kept confidential, but anonymised quotes may be published/presented.

2. **Background**
- Demographics: name, age, gender, country, centre name, rural or urban setting, government funded or non-government funded institution
- Job
  Q: What is your role in the surgical team?
  P: consultant/attending, fellow/registrar/resident, medical student, nurse
- Experience
  Q: Would you consider yourself experienced in minimal access surgery?
  P: robotic/laparoscopic, number of years, formal training/qualification.

3. **Robotics training**
- Q: *How did you train/are you training in robotics surgery?*
  P: fellowship; proctor; courses; simulation; accreditation.
- Q: How did your learning curve in robotics compare to laparoscopic surgery?
  P: Speed of acquisition of skills, haptic feedback, complications.

4. **Theatre efficiency**
- Impact on teamwork
  Q: *Based on your experience, how does robotic surgery affect team work? How does this differ from laparoscopic/open surgery?*
  P: theatre dynamics, situational awareness, communication, decision making, theatre turnover time, intra-operative complications/emergencies
- Impact on the healthcare professional
  Q: *What have been the positive/negative implications of robotic surgery for you?*
  P: *musculoskeletal injuries, fatigue, stress (mental/physical).*
- Impact on clinical outcomes for the patient
  Q: *In your experience, what have been the positive/negative impact of robotic surgery for your patients?*
  P: *blood loss, complications (intra/post-operative), anaesthetic time, length of hospital stay*
- *Strategies for implementing a robotics programme*
  Q: Describe the setup of your robotics service. What went well? What did not go so well? If you could do it again, what would you change?
  P: logistics, service structure
- *Facilitators/barriers to set up of a robotics programme*
  Q: What were the facilitators/barriers to set up? Why do you feel this way?
  P: cost, time, training, team, theatre space
- *Q: Are there service design factors that have contributed to operation duration/complications/conversion rates/theatre efficiency? Is so what are these?*
  P: peri-operative care, enhanced recovery, local geography

5. Surgical waste production

- *Q: Are you conscious of surgical waste production when performing robotic surgery?*
  P: single use versus multiple use equipment, equipment longevity for multiple use, carbon footprints – selection of equipment on this basis, green theatres, power off, national/local policy.
- *Q: Do you regularly review and rationalise your use on intra-operative equipment?*
  P: reusable versus single use, essential versus optional items to have ready on side, supplier engagement.
- *Q: Is your surgical equipment recycled?*
  P: repair or replace equipment, hospital policy.

Q - question; P – probe.

Final steps:

Thank the participant. Check whether they have remaining questions or comments about the topic
Reassurance about confidentiality and anonymity
Research team contact details should they want further information
