# Supplementary material for: Robotic Surgery from a Gynaecological Oncology Perspective: A Global Gynaecological Oncology Surgical Outcomes Collaborative Led Study (GO SOAR3)
Source: Diseases. 2025 Jan 6;13(1):9. doi: 10.3390/diseases13010009 (PMC11765035; doi:10.3390/diseases13010009)
Supplement: Supplementary file 1 [file diseases-13-00009-s001.zip › supplementary_tables_vF.pdf]

Table S1: Survey respondents per country

LMIC		HIC	
Botswana	1	Australia	4
Brazil	1	Austria	1
Egypt	1	Belgium	2
India	9	Canada	11
Iran	1	Denmark	2
Kazakhstan	1	France	5
Mexico	1	Germany	8
Russia	1	Greece	4
Tunisia	1	Hungary	1
Turkey	3	Ireland	1
Ukraine	2	Italy	19
Vietnam	1	Japan	1
		Luxembourg	1
		Netherlands	7
		Norway	1
		Portugal	3
		Romania	2
		Singapore	2
		Spain	8
		Sweden	8
		Switzerland	1
		Taiwan	1
		United Kingdom	25
		United States of America	11

LMIC – low and middle income country; HIC – high income country,

Table S2: Impact of robotic surgery on situational awareness in comparison to laparoscopic surgery

	Consultant (n=97, %)	Trainee (n=55, %)	P value
**Strongly agree**	8 (8.2%)	0 (0%)	0.151
**Agree**	27 (27.8%)	14 (25.5%)	
**neither agree or disagree**	18 (18.6%)	10 (18.2%)	
**Disagree**	28 (28.9%)	23 (41.8%)	
**Strongly disagree**	16 16.5%)	8 (14.5%)	

Survey question: In comparison to laparoscopic surgery, my intra-operative situational awareness (defined as the perception of elements in the environment, the comprehension of their meaning, and the projection of their status in the near future) with robotic surgery is reduced

Tables S3: Reasons for reduced situational awareness

	Consultant (n=35, %)	Trainee (n=14, %)	P value
**Surgeon sits at a console away from the patient and the rest of the operating room team**	28	11	1
**reduced visual perception**	24	12	0.297
**impaired communication with the operating room team**	20	7	0.755
**Other**	2	0	1

Table S4: Impact of lack of haptic feedback on robotic surgical ability in comparison to laparoscopic surgery

	Consultant (n=96, %)	Trainee (n=54, %)	P value
**Strongly agree**	6 (6.3%)	0 (0%)	0.333
**Agree**	21 (21.9%)	12 (22.2%)	
**neither agree or disagree**	16 (16.7%)	9 (16.7%)	
**Disagree**	31 (32.3%)	23 (42.6%)	
**Strongly disagree**	22 (22.9%)	10 (18.5%)	

Survey question: When compared to laparoscopic surgery, my surgical ability is negatively impacted with robotic surgery by lack of tactile information/haptic feedback

Table S5: Intra-operative complications robotic versus laparoscopic surgery

	Consultant (n=91, %)	Trainee (n=53, %)	P value
**Higher**	1 (1.1%)	0 (0%)	0.016
**somewhat higher**	1 (1.1%)	0 (0%)	
**the same**	58 (63.7%)	40 (75.5%)	
**somewhat lower**	4 (4.4%)	7 (13.2%)	
**lower**	27 (29.7%)	6 (11.3%)	

Survey question: In comparison to laparoscopic surgery, my intra-operative complications with robotic surgery are

Table S6: Impact of robotic versus laparoscopic surgery on physical fatigue

	Consultant (n=93, %)	Trainee (n=53, %)	P value
**Very much less physically tiring**	60 (64.5%)	38 (71.7%)	0.734
**somewhat less physically tiring**	25 (26.9%)	14 (26.4%)	
**as physically tiring**	5 (5.4%)	1 (1.9%)	
**somewhat more physically tiring**	2 (2.2%)	0 (0%)	
**very much more physically tiring**	1 (1.1%)	0 (0%)	

Survey question: In terms of physical fatigue, robotic surgery when compared to laparoscopic surgery is

Table S7: Impact of robotic versus laparoscopic surgery on mental fatigue

	Consultant (n=93, %)	Trainee (n=53, %)	P value
**Very much less mentally tiring**	16 (17.2%)	4 (7.5%)	0.143
**somewhat less mentally tiring**	33 (35.5%)	13 (24.5%)	
**as mentally tiring**	36 (38.7%)	29 (54.7%)	
**somewhat more mentally tiring**	6 (6.5%)	6 (11.3%)	
**very much more mentally tiring**	2 (2.2%)	1 (1.9%)	

Survey question: In terms of mental fatigue, robotic surgery when compared to laparoscopic surgery is

Table S8: Impact of robotic versus laparoscopic surgery on stress

	Consultant (n=92, %)	Trainee (n=53, %)	P value
**Very much less stressful**	15 (16.3%)	5 (9.4%)	0.034
**somewhat less stressful**	37 (40.2%)	12 (22.6%)	
**as stressful**	32 (34.8%)	32 (60.4%)	
**somewhat more stressful**	7 (7.6%)	3 (5.7%)	
**very much more stressful**	1 (1.1%)	1 (1.9%)	

Survey question: In terms of stress, robotic surgery when compared to laparoscopic surgery is

Table S9: Multiple regression analysis evaluating impact of variables on mental fatigue

Variable	OR	95% CI	p-value
Age	0.976	0.832 to 1.124	0.749
Gender	1.022	0.281 to 3.805	0.973
Job	0.492	0.111 to 2.069	0.335
Post grad experience	1.01	0.837 to 1.211	0.917
Laparoscopic surgical experience	1.034	0.891 to 1.22	0.677
Laparoscopic cases per annum	0.998	0.976 to 1.017	0.842
Robotic surgical experience	0.985	0.787 to 1.141	0.867
Robotic cases per annum	0.965	0.922 to 0.996	0.041

N=81. Very much more/somewhat more versus very much less/somewhat less.

Gender: male versus female. Job: consultant versus trainee. Age, post graduate experience, laparoscopic surgical experience (years): linear, laparoscopic cases per annum: linear, robotic surgical experience (years): linear, robotic cases per annum: linear.

Table S10: Multiple regression analysis evaluating impact of variables on stress

Variable	OR	95% CI	p-value
Age	0.819	0.608 to 1.027	0.127
Gender	9.328	1.334 to 196.923	0.056
Job	0.178	0.026 to 1.057	0.061
Post grad experience	1.179	0.946 to 1.524	0.163
Laparoscopic surgical experience	1.108	0.943 to 1.363	0.258
Laparoscopic cases per annum	1.008	0.986 to 1.029	0.439
Robotic surgical experience	1.016	0.822 to 1.175	0.850
Robotic cases per annum	0.967	0.924 to 0.995	0.038

N=81. Very much more/somewhat more versus very much less/somewhat less.

Gender: male versus female. Job: consultant versus trainee. Age, post graduate experience, laparoscopic surgical experience (years): linear, laparoscopic cases per annum: linear, robotic surgical experience (years): linear, robotic cases per annum: linear.

Table S11: Impact of robotic versus laparoscopic surgery on operating time

	Consultant (n=91, %)	Trainee (n=53, %)	p-value
**Strongly agree**	12 (13.2%)	6 (11.3%)	0.01
**Agree**	25 (27.5%)	29 (54.7%)	
**Neither agree or disagree**	16 (17.6%)	8 (15.1%)	
**Disagree**	27 (29.7%)	9 (17%)	
**Strongly disagree**	11 (12.1%)	1 (1.9%)	

Survey question: In terms of total operating time (time period includes docking as well as surgical procedure but excludes anaesthetic time), my robotic surgical cases take longer than laparoscopic surgery

Table S12: Reasons for increased operating time

	Consultant (n=37, %)	Trainee (n=35, %)	P value
**Surgeon speed slower with robotic surgery**	13	23	0.018
**increased time associated with set up and docking of ports**	32	35	0.054
**operating team less experienced with robotic surgery**	14	8	0.206

Table S13: Multiple regression analysis evaluating impact of variables on operating time

Variable	OR	95% CI	p-value
Age	1.019	0.902 to 1.165	0.773
Gender	1.772	0.674 to 4.874	0.254
Job	0.213	0.059 to 0.711	0.014
Post grad experience	0.995	0.873 to 1.122	0.933
Laparoscopic surgical experience	1.026	0.912 to 1.168	0.672
Laparoscopic cases per annum	1.006	0.993 to 1.02	0.338
Robotic surgical experience	0.897	0.778 to 1.007	0.098
Robotic cases per annum	0.969	0.947 to 0.987	0.003

N=120. Very much more/somewhat more versus very much less/somewhat less.

Gender: male versus female. Job: consultant versus trainee. Age, post graduate experience, laparoscopic surgical experience (years): linear, laparoscopic cases per annum: linear, robotic surgical experience (years): linear, robotic cases per annum: linear.

Table S14: Impact of robotic versus laparoscopic surgery on rate of conversion to open surgery

	Consultant (n=91, %)	Trainee (n=53, %)	p-value
**Higher**	2 (2.2%)	0 (0%)	0.176
**Somewhat higher**	1 (1.1%)	0 (0%)	
**the same**	61 (67%)	42 (79.2%)	
**somewhat lower**	9 (9.9%)	7 (13.2%)	
**lower**	18 (19.8%)	4 (7.5%)	

Survey question: My rate of conversion to open surgery with robotic surgery when compared to laparoscopic surgery is

Table S15: Impact of robotic versus laparoscopic surgery on learning curve

	**Consultant (n=97, %)**	**Trainee (n=55, %)**	**p-value**
**Much steeper**	3 (3.1%)	1 (1.8%)	0.039
**Steeper**	6 (6.2%)	1 (1.8%)	
**The same**	8 (8.2%)	4 (7.3%)	
**Less steep**	32 (33%)	32 (58.2%)	
**Much less steep**	48 (49.5%)	17 (30.9%)	

Survey question: My learning curve for robotic surgery when compared to laparoscopic surgery was

Table S16: Reasons survey respondents prefer robotic surgery over laparoscopic surgery

	Consultant (n=86, %)	Trainee (n=50, %)	p-value
**Less physical exhaustion for the surgeon**	69, (80.2%)	42, (84%)	0.652
**less mental exhaustion for the surgeon**	32, (37.2%)	8, (16%)	0.011
**enhanced visualisation in 3D of the surgical field**	72, (83.7%)	41, (82%)	0.816
**greater surgical dexterity**	66, (76.7%)	41, (82%)	0.522
**greater surgical precision**	68, (79.1%)	46, (92%)	0.056
**less reliance on a surgical assistant**	53, (61.6%)	32, (64%)	0.855
**fewer distractions when operating**	20, (23.3%)	7, (14%)	0.265

Table S17: Reasons survey respondents do not prefer robotic surgery over laparoscopic surgery

	**Consultant (n=11, %)**	**Trainee (n=5, %)**	**p-value**
**Less experience with robotic surgery**	6, (54.5%)	2 (40%)	1
**negative impact on communication in the operating theatre**	2 (18.2%)	1 (20%)	1
**negative impact on team work in the operating theatre**	2 (18.2%)	1 (20%)	1
**lack of tactile information/haptic feedback**	4 (36.4%)	2 (40%)	1
**spatial configuration of equipment in the operating theatre**	2 (18.2%)	1 (20%)	1
